# Multidimensional Motor Phenotype Characterization in Children with Joubert Syndrome: A Cross-Sectional Cohort Study

**DOI:** 10.3390/jcm15093221

**Published:** 2026-04-23

**Authors:** Łukasz Mański, Aleksandra Moluszys, Anna Góra, Eliza Wasilewska, Agnieszka Rosa, Jan Szumlicki, Krzysztof Szczałuba, Krystyna Szymańska, Jolanta Wierzba

**Affiliations:** 1 Gdansk Medical Academy of Applied Sciences, 80-335 Gdansk, Poland; aleksandra.moluszys@gam.edu.pl (A.M.);; 2 Department of Allergology, Medical University of Gdansk, 80-210 Gdansk, Poland; 3 Center of Excellence for Rare and Undiagnosed Disorders, Medical University of Warsaw, 02-091 Warsaw, Poland; agarosa@vp.pl (A.R.); krzysztof.szczaluba@wum.edu.pl (K.S.); 4 Department of Pediatric Neurology and Rare Diseases, Medical University of Warsaw, 02-091 Warsaw, Poland; krystyna.szymanska@uckwum.pl; 5 Department of Internal and Pediatric Nursing, Institute of Nursing and Midwifery, Medical University Gdansk, 80-208 Gdansk, Poland

**Keywords:** Joubert syndrome, cerebellar disorders, motor phenotype, postural control, sacral slope, thoracoabdominal configuration, physiotherapy assessment, axial organization

## Abstract

**Background:** Joubert syndrome (JS) is a rare neurodevelopmental ciliopathy characterized by cerebellar and brainstem malformation and heterogeneous motor outcomes. Although global developmental delay is well described, integrated clinical characterization combining functional and structural domains remains limited. The aim of this exploratory study was to describe a multidimensional motor phenotype in children with JS and to explore associations between selected functional and structural parameters. **Methods:** A prospective cross-sectional cohort study was conducted in 25 children with MRI-confirmed JS (aged 2–16 years). Gross motor performance was assessed using the GMFM-88, and postural control was evaluated with the modified Brief Ataxia Rating Scale (mBARS). Measured musculoskeletal parameters included joint range of motion and sacral slope as an indicator of sagittal pelvic alignment. Thoracoabdominal configuration was assessed using angular and anthropometric measurements. Associations between predefined functional and structural variables were explored using Spearman’s rank correlation coefficients. Analyses were exploratory and hypothesis-generating and were not adjusted for multiple comparisons. **Results:** Marked inter-individual variability was observed across functional and structural domains. A moderate negative correlation was identified between GMFM-88 (Gross Motor Function Measure-88) and mBARS (modified Brief Ataxia Rating Scale) scores (ρ = −0.512, *p*-value = 0.007). Sacral slope demonstrated statistically significant associations with hip extension, ankle dorsiflexion, chest and abdominal circumference, and sternoclavicular alignment (all *p*-value < 0.05). No significant correlations were detected between gross motor performance and isolated structural parameters. Intra-rater reliability of selected measurements was high (ICC range 0.939–0.999). **Conclusions:** This exploratory, hypothesis-generating study demonstrates multidimensional variability in motor organization encompassing functional, postural, and structural domains. Gross motor performance appears more closely related to postural control than to isolated peripheral structural measures within this exploratory framework. Structural parameters exhibit internal statistical co-variation but do not independently determine functional capacity. These findings support the value of integrated multidomain physiotherapy assessment in rare cerebellar neurodevelopmental disorders.

## 1. Introduction

Joubert syndrome (JS) is a rare neurodevelopmental ciliopathy characterized by malformation of the cerebellar vermis and brainstem, producing the characteristic molar tooth sign on neuroimaging [1,2]. JS represents a heterogeneous group of disorders that may occur as an isolated neurodevelopmental condition or as part of a broader multisystem phenotype. It is most inherited in an autosomal recessive manner and has been associated with mutations in several genes encoding proteins of the primary cilium, such as *CEP290*, *TMEM67*, and *AHI1* (genes encoding ciliary proteins) [3,4,5]. Clinically, these structural abnormalities are associated with hypotonia, ataxia, and delayed motor development [5,6,7]. Multisystem involvement is common and may include ocular, renal, hepatic, and respiratory manifestations, reflecting the systemic nature of ciliopathy-related dysfunction [8,9]. The estimated prevalence of JS ranges from approximately 1 in 80,000 to 1 in 100,000 live births worldwide, although precise epidemiological data remain limited [10]. Country-specific prevalence of Joubert syndrome, including data from Poland, is not well established due to limited epidemiological data, lack of dedicated registries, and potential underdiagnosis of rare diseases within the Polish healthcare system [8,11,12].

Motor outcomes in JS are heterogeneous and vary according to genetic background, neuroanatomical involvement, and developmental trajectory [1,6,13]. Functional motor performance is typically described using global developmental measures; however, less attention has been given to the combined assessment of motor performance, postural control, musculoskeletal organization, and thoracoabdominal configuration within a single structured framework [13,14]. This gap is particularly relevant from a rehabilitation perspective, where integrated assessment of functional performance, postural control, and structural organization may inform individualized therapeutic strategies. Despite increasing clinical recognition of JS, multidimensional physiotherapy-based characterization remains underexplored [3,10].

Despite increasing recognition of the genetic and neuroimaging spectrum of JS, standardized clinical characterization of the motor phenotype remains limited. Most available reports describe developmental delay in broad terms, without systematic integration of postural control, axial alignment, and structural organization. Consequently, relationships between functional performance and biomechanical configuration remain insufficiently explored in pediatric cohorts with JS [15].

Postural control and axial alignment contribute to functional motor performance, and trunk organization may be particularly relevant in neurodevelopmental conditions affecting cerebellar and brainstem structures [16,17,18]. In such conditions, differences in central motor regulation may be accompanied by variations in postural organization and musculoskeletal or thoracoabdominal configuration [19,20,21]. Although advances in genetic and neuroimaging research have improved understanding of the underlying pathophysiology of JS, clinical descriptions of motor phenotype have primarily focused on global developmental outcomes. Structural characteristics and their relationships with functional performance have been less frequently examined within an integrated framework. A multidimensional physiotherapy-based assessment integrating functional and structural domains may enable more comprehensive characterization of the motor phenotype and support rehabilitation planning [22,23]. The multidimensional assessment framework applied in this study is conceptually aligned with the International Classification of Functioning, Disability and Health (ICF), as it integrates domains of body structure, body function, and activity within a single clinical model [24].

Accordingly, the aim of this exploratory study was to describe functional motor performance, postural control, musculoskeletal organization, and thoracoabdominal configuration in children with JS and to explore associations between selected functional and structural parameters. Given the exploratory and hypothesis-generating design, no formal a priori null or alternative hypotheses were specified.

To our knowledge, few studies have attempted to integrate gross motor performance, postural control, sagittal pelvic alignment, and thoracoabdominal configuration within a single structured physiotherapy-based assessment framework in children with JS. Recent studies have emphasized the need for multidimensional assessment approaches in JS, highlighting that global motor scales may not fully capture axial, postural, and respiratory-related functional domains [25,26]. Emerging evidence also suggests that motor phenotype in JS reflects complex interactions between cerebellar, postural, and respiratory systems rather than isolated motor deficits [27].

## 2. Materials and Methods

### 2.1. Study Design

The study was designed as a prospective cross-sectional cohort investigation, with each participant undergoing a single standardized assessment session without a control group. Each participant completed a single assessment session. The study did not include randomization or blinding procedures. Evaluations were conducted over a four-month period (June–September 2025) in two environments: a dedicated assessment session organized within a national family meeting for children with JS and their caregivers, and a pediatric neurorehabilitation outpatient setting (Gdańsk, Poland). All evaluations were conducted under consistent conditions according to a predefined assessment protocol. The sample size was feasibility-based and reflected the availability of children with confirmed JS during the study period. Given the exploratory nature of the study, analyses focused on characterization of functional and structural features and exploratory assessment of associations between selected variables.

### 2.2. Participants

The study included 25 children with a confirmed diagnosis of JS. Both isolated forms and JS-related disorders were represented, reflecting the clinical and genetic heterogeneity of the condition. Diagnosis was established by experienced pediatric neurologists based on magnetic resonance imaging (MRI) demonstrating the molar tooth sign, with molecular genetic testing performed as part of routine clinical care when available. Brain MRI was performed using standard clinical protocols on 1.5T or 3T scanners, including T1-weighted, T2-weighted, and FLAIR sequences. Imaging data were obtained as part of routine diagnostic procedures and were reviewed by experienced pediatric neurologists.

Eligible participants were children aged 2–16 years with clinical stability allowing completion of the assessment protocol. Exclusion criteria included severe cardiorespiratory instability, acute orthopedic injury, orthopedic surgery within the preceding six months, or inability to complete any component of the assessment. These criteria were applied to ensure measurement reliability and minimize potential confounding effects on motor and respiratory parameters.

Participants were enrolled through collaboration with a national parent organization for families of children with JS. Assessments were conducted in Poland in two settings: during a nationwide family meeting and at a pediatric neurorehabilitation outpatient clinic in Gdańsk. Most participants (n = 22) were evaluated during the family meeting, while three children were assessed in the outpatient setting. This recruitment approach enabled inclusion of participants from multiple regions within a limited timeframe. All assessments followed a standardized protocol and were performed under consistent conditions. Enrollment was based on availability during the study period, and all eligible participants were invited to take part. The recruitment context may have introduced selection bias toward families more actively engaged in care or rehabilitation services. Demographic and anthropometric data, including age, sex, height, and body weight, were collected at the time of assessment. Participants were managed within multidisciplinary clinical care, including pediatric neurologists and other relevant specialists as required.

### 2.3. Ethical Considerations

The research protocol complied with the Declaration of Helsinki and was approved by the Independent Bioethics Committee for Scientific Research at the Medical University of Gdansk (approval No. KB 194/2025; 9 May 2025). Prior to inclusion, written informed consent was obtained from parents or legal guardians. Data were anonymized before being included in the analysis.

### 2.4. Parent-Reported Clinical Questionnaire

Additional clinical information was obtained using a structured parent-reported questionnaire developed for this study. The questionnaire collected descriptive data regarding medical history, organ involvement (ocular, renal, hepatic), respiratory symptoms, sleep-related breathing disorders, visual abnormalities, and other systemic comorbidities. The questionnaire was intended as a descriptive tool to support characterization of the multisystem clinical phenotype rather than as a diagnostic instrument. It was completed by parents or legal guardians prior to or during the assessment session. Data derived from the questionnaire were used exclusively for descriptive cohort characterization and were not included in correlation or inferential analyses. As the questionnaire was parent-reported and not independently verified against medical records, responses were considered descriptive and may be subject to reporting bias.

### 2.5. Functional Assessment Framework

A multidimensional assessment framework was applied to characterize functional organization in children with JS. The protocol was designed a priori to capture four complementary domains: gross motor performance, postural and axial control, musculoskeletal organization, and thoracoabdominal configuration. This approach enabled integrated characterization of functional phenotype beyond single global motor scores. All assessments were conducted by the same experienced pediatric physiotherapist. The examiner was not blinded to diagnosis.

### 2.6. Gross Motor and Postural Control Assessment

Gross motor function was evaluated using the GMFM-88, with scores calculated according to established guidelines. Postural control and coordination were examined using the modified Brief Ataxia Rating Scale (mBARS), selected for its relevance to cerebellar dysfunction. Although mBARS has not been specifically validated for JS, it was selected based on its construct relevance to cerebellar-related motor dysfunction, particularly axial control and coordination, which are consistently described features of the JS motor phenotype. The scale was used to characterize postural and coordination performance within the cohort rather than as a diagnostic instrument. Total GMFM-88 and mBARS scores were used for statistical analyses.

### 2.7. Postural and Musculoskeletal Assessment

Musculoskeletal assessment included evaluation of hip, shoulder, and ankle range of motion and measurement of the sacral slope angle as an indicator of pelvic alignment in the sagittal plane. Joint range of motion was measured using a handheld goniometer based on predefined anatomical landmarks and consistent positioning across participants. Bilateral measurements were obtained for descriptive purposes. Sacral slope was measured using a mechanical inclinometer positioned over the sacral surface. All angular measurements were performed twice within the same session by the same examiner, and the mean value was used for analysis. Selected parameters were additionally included in intra-rater reliability analysis. Sacral slope was selected as a clinically accessible and reproducible proxy of sagittal pelvic alignment, reflecting axial orientation within the postural system and its potential relationship with trunk organization.

### 2.8. Thoracoabdominal Configuration and Respiratory Measures

Thoracoabdominal configuration was assessed using anthropometric and angular measurements. Chest and abdominal circumferences were measured at predefined anatomical landmarks using a flexible, non-elastic measuring tape. Thoracic expansion during quiet breathing was calculated as the difference between end-inspiratory and end-expiratory circumferential measurements obtained during spontaneous breathing. Thoracic angular parameters were measured using a handheld universal goniometer in the supine position on a flat examination surface, with the head in neutral alignment and upper limbs resting alongside the body. Respiratory rate was recorded manually over a 60 s period during quiet breathing following a short rest phase. All thoracoabdominal measurements were performed twice within the same session by the same examiner, and the mean value was used for analysis. Surface-based angular parameters were applied as standardized geometric descriptors within a predefined clinical protocol. To date, widely established age-stratified normative reference values for these thoracoabdominal angular measures in pediatric populations are not available. Accordingly, these parameters were interpreted descriptively as indicators of trunk configuration within the cohort rather than as normative or diagnostic measures. Thoracoabdominal parameters were included to capture axial configuration relevant to the integration of postural and respiratory function, which are closely linked in cerebellar and brainstem-related disorders.

### 2.9. Statistical Analysis

Data analysis was conducted using Jamovi software (version 2.7.12), with continuous variables presented as mean ± SD or median and range where appropriate. Normality was assessed using the Shapiro–Wilk test. Associations between predefined functional and structural parameters were explored using Spearman’s rank correlation coefficients. Analyses were considered exploratory and were not adjusted for multiple comparisons. A two-tailed *p*-value < 0.05 was considered statistically significant. Correlation analyses were exploratory and conducted within the predefined multidimensional assessment framework. Intra-rater reliability was evaluated using the intraclass correlation coefficient (ICC (3,1); two-way mixed-effects model, absolute agreement). Measurement error was quantified using the standard error of measurement (SEM) and minimal detectable change at the 95% confidence level (MDC95). Agreement was additionally examined using Bland–Altman analysis; detailed plots are available in the Supplementary Materials (Figures S1–S3). The complete predefined correlation matrix is provided in Supplementary Table S1 to ensure analytical transparency. Given the exploratory nature and limited sample size, correction for multiple comparisons was not applied to avoid excessive type II error, and results should be interpreted as hypothesis-generating.

**Figure 1:**
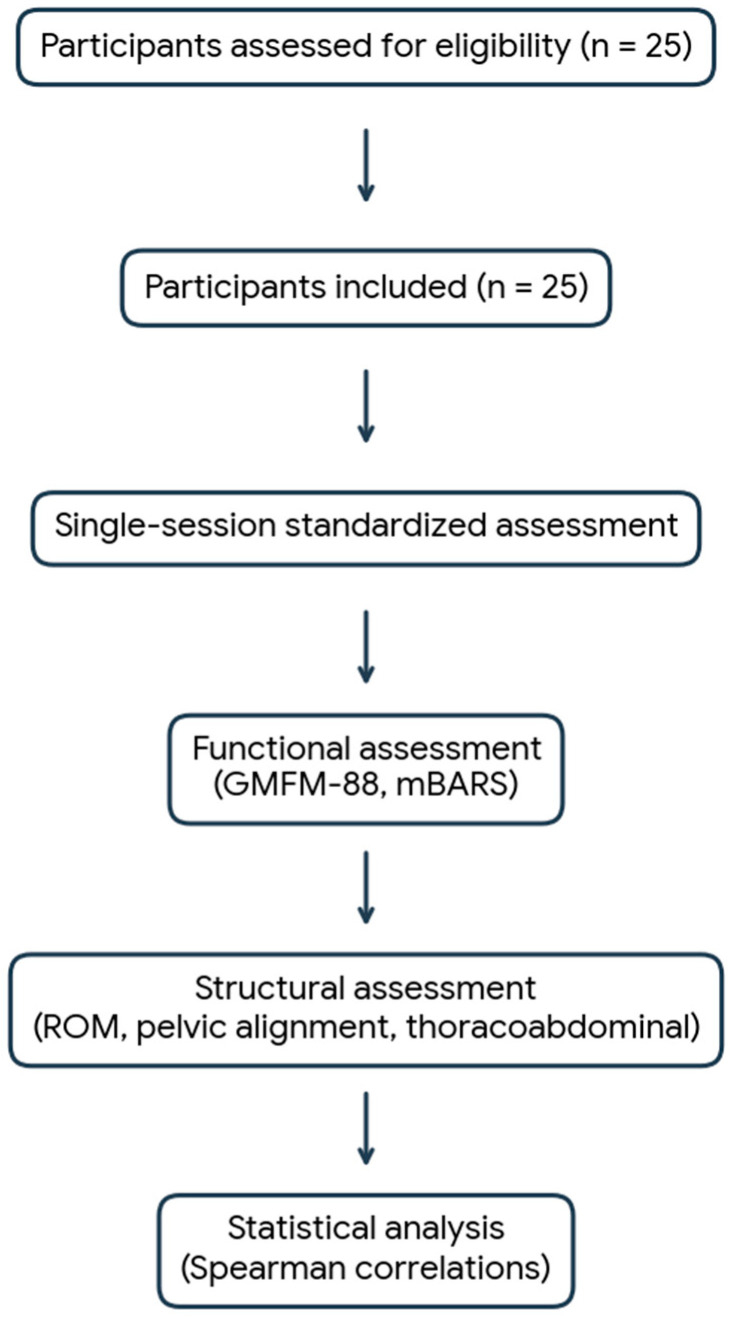
Flowchart of the study design, recruitment process, and assessment procedure. Abbreviations: GMFM-88, Gross Motor Function Measure-88; mBARS, modified Brief Ataxia Rating Scale.

The study workflow, including participant recruitment and assessment procedures, is summarized in Figure 1.

## 3. Results

The Results section is organized to first present general cohort characteristics, followed by functional, structural, and integrated multidimensional analyses reflecting the study’s physiotherapy-oriented framework.

### 3.1. Cohort Characteristics and Multisystem Features

The cohort included 25 children (mean age 7.8 ± 4.0 years, range 2–16), including 16 males (64%) and 9 females (36%). All participants had MRI-confirmed JS; molecular genetic confirmation was available in 22 (88%). Three participants (12%) had undergone kidney transplantation, and one (4%) had prior scoliosis surgery. Ocular findings included nystagmus in 16 participants (64%), strabismus in 9 (36%), refractive errors in 8 (32%), oculomotor apraxia in 3 (12%), retinal dystrophy in 3 (12%), coloboma in 1 (4%), ptosis in 1 (4%), and severe visual impairment in 1 (4%). Renal involvement was reported in 8 participants (32%), including cystic kidney disease in 3 (12%) and chronic kidney disease in 1 (4%). Liver involvement was reported in 3 participants (12%), and arterial hypertension in 1 (4%). Respiratory features included frequent respiratory infections in 12 participants (48%), snoring in 8 (32%), abnormal breathing sounds in 7 (28%), sleep apnea in 5 (20%), cough in 4 (16%), hyperventilation in 2 (8%), and other respiratory dysregulation symptoms in 4 (16%).

Participants frequently presented with multiple concurrent clinical features; therefore, categories were not mutually exclusive. Genetic, clinical, and multisystem characteristics are summarized in Table 1, providing an integrated overview of the cohort.

### 3.2. Overview of Developmental Milestones and Rehabilitation Exposure

Developmental milestones and rehabilitation exposure are summarized in Table 2. Head control and independent sitting were achieved by all participants. Independent standing was achieved by 21 participants (84%), while 4 (16%) did not achieve independent standing. Independent walking without assistance was achieved by 23 participants (92%), and 2 (8%) did not achieve independent walking.

Neurodevelopmental treatment (NDT Bobath) was reported in 23 participants (92%), speech therapy in 22 (88%), and sensory integration therapy in 21 (84%). Thirteen participants (52%) received ≥5 therapist-led sessions per week, 9 (36%) received 3–4 sessions per week, and 3 (12%) received ≤2 sessions per week. Daily home therapy was reported in 6 participants (24%), while 14 (56%) did not report regular home therapy. Most participants received more than one rehabilitation modality concurrently, reflecting a multimodal therapeutic approach.

### 3.3. Gross Motor Function and Postural Control

Gross motor and postural control data are presented in Table 3. The mean GMFM-88 total score was 58.4 ± 29.5%, with a median of 78.8% and a range of 3.0–89.4%. The mean mBARS total score was 14.4 ± 9.47 points, with a median of 13 and a range of 2–30 points. Shapiro–Wilk testing indicated non-normal distribution for both GMFM-88 (W = 0.796, *p*-value < 0.001) and mBARS (W = 0.897, *p*-value = 0.015), reflecting substantial inter-individual variability within the cohort.

#### Functional Distribution of Motor Performance

To facilitate clinical interpretation of the observed variability, an approximate distribution of motor performance and postural control levels was examined. Based on GMFM-88 scores, participants demonstrated a broad functional spectrum, ranging from very low (<20%) to high (>80%) motor performance. A substantial proportion of the cohort achieved moderate-to-high levels of gross motor function, while a smaller subgroup presented with markedly reduced motor capacity. Similarly, mBARS scores indicated a wide distribution of postural and coordination impairment, from mild to severe. Some participants demonstrated relatively preserved postural control, whereas others exhibited pronounced ataxia and coordination deficits.

Overall, these findings illustrate that children with JS in this cohort do not form a homogeneous functional group but rather span a continuum from near-functional independence to significant motor impairment.

### 3.4. Musculoskeletal and Postural Organization

Musculoskeletal and postural parameters are summarized in Table 4. Bilateral shoulder external rotation demonstrated a mean of 98.4 ± 27.4 degrees and a median of 110 degrees. Hip external rotation had means of 65.6 ± 23.3 degrees (left) and 63.0 ± 26.6 degrees (right). Hip extension had means of 15.6 ± 8.2 degrees (left) and 16.0 ± 7.6 degrees (right). Ankle dorsiflexion had means of 4.2 ± 7.4 degrees (left) and 5.7 ± 8.0 degrees (right). Sacral slope had a mean of 16.9 ± 3.8 degrees and a median of 16 degrees. Normality assessment using the Shapiro–Wilk test revealed a non-normal distribution of the analyzed variables (W = 0.907).

### 3.5. Thoracoabdominal Configuration and Respiratory Characteristics

Thoracoabdominal and respiratory parameters are presented in Table 5. Mean sternoclavicular angles were 122 ± 11.2 degrees (right) and 125 ± 9.0 degrees (left). The mean subcostal angle was 119 ± 16.7 degrees. Chest and abdominal circumferences demonstrated mean values of 65.0 ± 11.1 cm and 62.0 ± 11.1 cm, respectively. Thoracic expansion during quiet breathing had a mean of 1.9 ± 1.3 cm. Respiratory rate averaged 17.2 ± 6.1 breaths per minute. Shapiro–Wilk testing indicated non-normal distribution for chest circumference, abdominal circumference, and thoracic expansion, whereas sternoclavicular angles, subcostal angle, and respiratory rate did not significantly deviate from normal distribution.

### 3.6. Associations Between Functional and Structural Parameters

Associations between functional and structural parameters were examined using Spearman’s rank correlation coefficients. Statistically significant correlations are presented in Table 6, and the complete correlation matrix is provided in Supplementary Table S1. A moderate negative correlation was observed between GMFM-88 and mBARS total scores (ρ = −0.512, *p*-value = 0.007). Sacral slope demonstrated negative correlations with hip extension (right: ρ = −0.614, *p*-value = 0.001; left: ρ = −0.629, *p*-value < 0.001) and ankle dorsiflexion (right: ρ = −0.464, *p*-value = 0.019; left: ρ = −0.456, *p*-value = 0.022). Positive correlations were observed between sacral slope and chest circumference (ρ = 0.724, *p*-value < 0.001), abdominal circumference (ρ = 0.530, *p*-value = 0.006), and left sternoclavicular angle (ρ = 0.457, *p*-value = 0.021). No additional correlations reached statistical significance.

### 3.7. Intra-Rater Reliability

Intra-rater reliability of selected measurements was evaluated using ICC (3,1), which values ranged from 0.939 to 0.999 (all *p*-value < 0.001). For sacral slope, the mean difference between repeated measurements was 0.88° (95% limits of agreement: −1.10° to 2.86°; *p*-value = 0.592 for proportional bias). For thoracic expansion, the mean difference was 0.72 cm (95% limits of agreement: −1.71 to 3.15 cm; *p*-value = 0.32). For ankle dorsiflexion (right), the mean difference was 0.28° (95% limits of agreement: −1.05° to 1.61°; *p*-value = 0.005). SEM and MDC95 values are presented in Table 7.

## 4. Discussion

### 4.1. Multidimensional Characterization of the Motor Phenotype

The main findings indicate that motor performance is more closely associated with postural control than with isolated structural measures, while structural parameters demonstrate internal co-variation without direct linkage to functional capacity. This approach differs from previous studies, which have primarily focused on neuroimaging, genetic subtypes, or global developmental outcomes, without systematically examining relationships between functional and structural domains within a physiotherapy-oriented framework.

The present study provides a structured multidimensional characterization of motor phenotype in children with JS, integrating gross motor performance, postural control, musculoskeletal organization, pelvic alignment, and thoracoabdominal configuration within a single physiotherapy-based assessment framework. Marked inter-individual differences were observed across all assessed domains, encompassing functional motor capacity, coordination, joint range of motion, sagittal pelvic orientation, and trunk configuration. This pattern is consistent with previous reports describing clinical heterogeneity in JS, which has been linked to variability in cerebellar and brainstem involvement as well as developmental trajectories [1,8]. Most prior investigations have focused primarily on neuroimaging findings, genetic subtypes, or global developmental milestones [7,28]. In contrast, the present analysis examined functional and structural domains concurrently, enabling observation of relational patterns across systems. In this context, motor presentation in JS may reflect multidimensional differences in axial and appendicular organization, potentially corresponding to distributed cerebellar-brainstem network involvement, rather than being characterized solely by delayed milestone acquisition [29,30,31]. The cohort was clinically heterogeneous, with multisystem involvement and variable rehabilitation exposure documented across participants. This recruitment context may also reflect a cohort with higher rehabilitation exposure and caregiver engagement, which could influence functional outcomes. Developmental milestone acquisition and therapy intensity varied considerably, reflecting the broad clinical spectrum of JS. Although these factors were not analyzed as modifiers of functional outcomes, they may contribute to the substantial inter-individual variability observed across domains. Consequently, the present findings should be interpreted within the context of a clinically diverse cohort rather than a phenotypically uniform group. Given the wide age range of the cohort, developmental stage-related differences in motor control, postural organization, and respiratory mechanics may have contributed to the observed variability. The absence of a control group further limits the ability to distinguish syndrome-specific patterns from broader neurodevelopmental variability. These maturational factors should be considered when interpreting cross-sectional associations within a heterogeneous pediatric population. The distribution of functional scores further highlights the clinical heterogeneity of JS. Children within the cohort ranged from those achieving relatively high levels of functional independence to those with substantial motor limitations. This variability is clinically meaningful, as it reflects differences not only in motor performance but also in daily activity, autonomy, and rehabilitation needs. From a practical perspective, these findings suggest that children with JS cannot be adequately described using a single functional profile, and individualized assessment remains essential for clinical decision-making.

### 4.2. Functional Motor Performance and Postural Control

A moderate negative association was observed between GMFM-88 and mBARS scores, indicating that lower gross motor performance was accompanied by greater impairment in postural control and coordination. This association is consistent with the established role of cerebellar circuits in integrating proprioceptive, vestibular, and visual inputs to generate coordinated motor output and anticipatory postural adjustments [32,33]. Structural abnormalities of the cerebellar vermis and brainstem, characteristic of JS, are known to affect these integrative processes [1]. Trunk control has been identified as an important component of gross motor performance in pediatric neurological populations [34,35]. Within this context, the observed relationship may be compatible with the contribution of centrally mediated postural regulation to functional motor expression. However, given the cross-sectional design, this finding should be interpreted as associative rather than causal. Therefore, mBARS results should be interpreted as descriptive indicators of coordination rather than validated outcome measures in this population. No causal or mechanistic inference can be drawn from these associations.

### 4.3. Measurement Reliability and Agreement

High intra-rater ICC (3,1) values indicate strong within-session consistency of repeated measurements performed by the same examiner. Bland–Altman analysis demonstrated small mean differences and relatively narrow limits of agreement for key measures, including sacral slope and thoracic expansion. No evidence of proportional bias was detected for these parameters. Although ankle dorsiflexion showed a statistically significant difference between repeated measures, the magnitude of this difference was small relative to the observed inter-individual variability. From a measurement perspective, this finding may reflect minor systematic variation rather than substantial instability of the parameter. Overall, the reliability analyses suggest that the variability identified across participants is unlikely to be explained solely by measurement error. In the context of rare pediatric conditions, where standardized structural assessment approaches remain limited, documentation of measurement agreement enhances the interpretability of descriptive findings [36]. Inter-rater reliability was not assessed in the present study and therefore cannot be inferred.

### 4.4. Structural Organization Within the Axial–Appendicular System

Exploratory correlation analyses identified statistically significant associations among selected structural parameters, including pelvic orientation, hip extension, ankle dorsiflexion, and thoracoabdominal measures. These findings do not indicate a single dominant structural determinant but instead suggest co-variation across components of the axial–appendicular system. Pelvic orientation represents one component of sagittal alignment and may reflect broader aspects of postural configuration [37,38]. From a neurobiomechanical perspective, postural alignment is understood to emerge from interaction between neural regulation and musculoskeletal mechanics [32,39]. In this conceptual model, the pattern of associations observed in the present study may be compatible with integrative models of postural control [27]. However, these correlations do not establish hierarchical organization or mechanistic linkage and should be interpreted strictly within an exploratory analytical context.

### 4.5. Thoracoabdominal Configuration and Axial Regulation

Thoracoabdominal parameters varied across participants, including sternoclavicular alignment, chest and abdominal circumferences, and thoracic expansion. Cerebellar and brainstem structures are involved in coordination of both postural and respiratory functions through distributed neural networks integrating axial motor output and respiratory rhythm generation [40,41]. The trunk functions as an integrated biomechanical and neurophysiological unit in which respiratory and postural systems interact continuously. The diaphragm and deep trunk musculature contribute to ventilation as well as spinal stabilization through modulation of intra-abdominal pressure and coordinated activation patterns [42,43]. Alterations in central motor control associated with cerebellar and brainstem involvement in JS have been described in relation to axial coordination and respiratory regulation [43,44]. In this broader neurophysiological context, variability in thoracoabdominal configuration observed in the present cohort may be compatible with differences in axial motor regulation. However, the current cross-sectional data do not allow conclusions regarding directionality, causality, or functional implications of these structural findings.

### 4.6. Functional-Structural Dissociation

**Figure 2:**
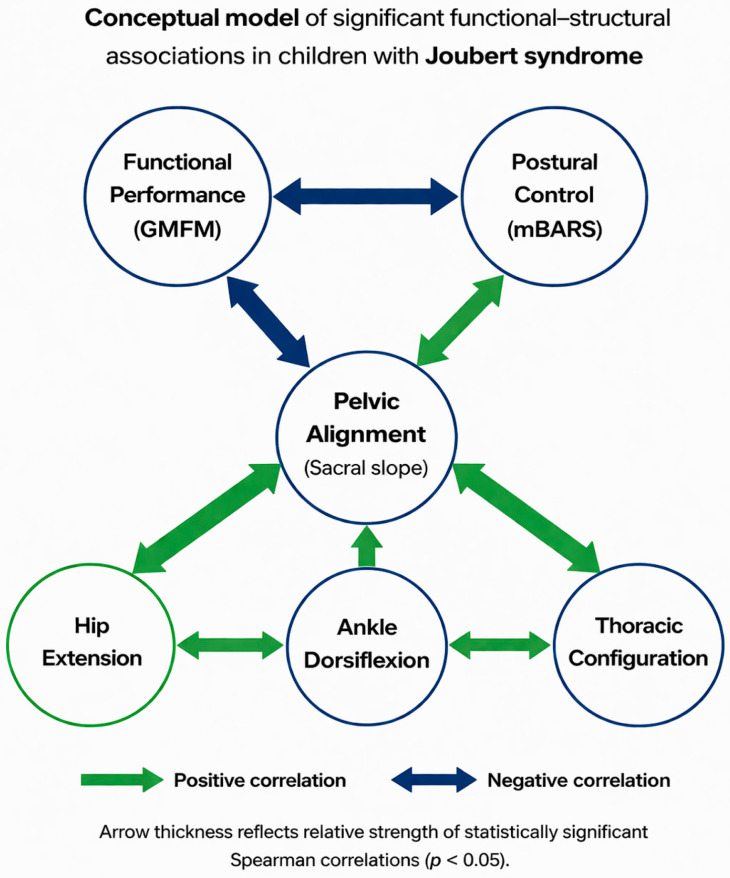
Conceptual summary of statistically significant associations between functional and structural parameters in children with JS (n = 25). Note: Green arrows indicate positive correlations, and blue arrows indicate negative correlations. Arrow thickness reflects the relative magnitude of statistically significant Spearman’s rank correlation coefficients (*p*-value < 0.05). Only associations reaching statistical significance within the predefined analytical framework are displayed. The model represents statistical co-variation observed within this cohort and does not imply causal relationships, mechanistic coupling, directional effects, or hierarchical organization between variables.

Importantly, functional motor performance was not significantly associated with isolated musculoskeletal or thoracoabdominal parameters in the present cohort. This pattern may indicate that single structural measures do not directly correspond to gross motor capacity within this cross-sectional framework. In contrast, functional performance was statistically associated with postural control and coordination, as reflected by the relationship between GMFM-88 and mBARS scores. Previous studies in cerebellar disorders have emphasized that motor impairment is frequently related to alterations in timing, coordination, and sensorimotor integration rather than to isolated peripheral biomechanical constraints [32,45]. Experimental and clinical investigations have demonstrated that cerebellar dysfunction affects predictive control, error correction, and anticipatory postural adjustments, processes that influence movement quality independently of joint range of motion or static alignment [27,46,47]. In this context, the absence of significant associations between GMFM-88 scores and individual structural parameters in the present study may be compatible with the central role of neural integration mechanisms in determining functional motor performance. In pediatric neurological populations, similar dissociations between structural alignment and functional capacity have been described, where joint range or segmental posture alone did not consistently predict gross motor outcomes [48,49]. These observations suggest that structural characteristics may coexist with functional impairment without directly determining performance level. Within a cerebellar framework, structural features may coexist with altered motor control without necessarily constituting primary determinants of functional limitation. Nevertheless, the absence of statistical association should be interpreted cautiously. The present study was exploratory, with a limited sample size and multiple comparisons not adjusted for type I error. It therefore cannot be concluded that structural parameters are unrelated to functional performance. Rather, the findings indicate that no direct linear relationships were detected within this cohort under the current analytical conditions. Importantly, the present results should not be interpreted as evidence of mechanistic coupling between structural alignment and neural motor control, but rather as statistical co-variation observed within a defined clinical cohort. A conceptual summary of the observed functional–structural associations is presented in Figure 2.

### 4.7. Methodological Considerations and Interpretative Caution

The present findings should be interpreted within the context of an exploratory cross-sectional design. Correlation analyses were not adjusted for multiple comparisons, and the ratio of analyzed variables to sample size increases the possibility of type I error. Consequently, statistically significant associations should be regarded as hypothesis-generating rather than confirmatory. The conceptual model presented in Figure 1 provides a structured visualization of statistically significant relationships identified within the predefined analytical framework. It does not imply causality, directionality, or predictive hierarchy among variables. Longitudinal and controlled studies are required to determine the stability and developmental relevance of the observed associations.

Additionally, the absence of correction for multiple comparisons increases the likelihood of type I error, particularly given the number of analyzed variables relative to sample size. Therefore, the reported statistically significant associations should be interpreted with caution and considered exploratory, requiring confirmation in larger and hypothesis-driven studies.

## 5. Clinical Implications

Given the exploratory design, the present findings may inform clinical assessment approaches in children with JS. The observed association between gross motor performance and postural control suggests that coordinated evaluation of functional ability and axial regulation may provide complementary information during physiotherapy assessment. The identified relationships among selected structural parameters indicate that pelvic orientation and thoracoabdominal configuration can be documented as part of a multidimensional clinical profile. However, the present data do not establish these measures as determinants of functional outcome nor as specific therapeutic targets. All assessment components were obtained using clinically accessible and low-cost tools. This feasibility may facilitate structured multidomain characterization in routine physiotherapy practice. Further longitudinal studies are necessary to determine whether integrating structural and functional measures contributes to prediction of developmental trajectories or response to intervention.

## 6. Study Limitations

This study has several limitations that should be considered when interpreting the findings. Importantly, the absence of a control group represents a key methodological limitation, as it precludes direct comparative interpretation and reduces the ability to distinguish syndrome-specific features from general neurodevelopmental variability.

The relatively small sample size, inherent to the rarity of JS, limits statistical power and restricts generalizability. The cross-sectional design precludes conclusions regarding temporal relationships, causality, or developmental trajectories. Correlation analyses were conducted within an exploratory framework and were not adjusted for multiple comparisons. Consequently, statistically significant associations should be interpreted as hypothesis-generating rather than confirmatory. The ratio of analyzed variables to sample size further increases the potential risk of type I error. The absence of a typically developing control group prevents direct comparison with normative values and limits differentiation between features potentially specific to JS and patterns that may also occur in other neurodevelopmental conditions. Additionally, postural and thoracoabdominal parameters were analyzed without systematic comparison to age-matched normative data, restricting interpretation to within-cohort characterization. These measures should therefore be interpreted as relative within-cohort descriptors rather than absolute biomechanical indicators.

The wide age range of participants introduces developmental heterogeneity, as axial control, postural organization, and respiratory mechanics undergo substantial maturational changes across childhood. Age-related variability may therefore have influenced the observed patterns and reduced comparability between participants. All assessments were conducted by a single non-blinded examiner. Although intra-rater reliability was evaluated, inter-rater reliability was not assessed and cannot be inferred. Measurements were obtained using clinically accessible tools rather than instrumented biomechanical systems, and surface-based thoracoabdominal angular parameters are not yet supported by widely established pediatric normative databases. These measures do not capture dynamic motor behavior or three-dimensional biomechanical interactions and should therefore be interpreted within a simplified structural framework. In addition, bilateral joint values were retained for descriptive transparency. Although this approach allowed comprehensive structural characterization, it increased the number of analyzed variables relative to sample size and may have contributed to statistical instability. Future studies with larger cohorts should consider dimensional reduction strategies to enhance robustness of analytical models.

Recruitment through voluntary participation, including partial recruitment during a national family meeting, may introduce selection bias and limit external validity. The cohort also included genetically heterogeneous subtypes of JS. Given the sample size, genotype-phenotype associations could not be explored, and genetic variability may have contributed to observed functional heterogeneity. Taken together, these limitations indicate that the findings are exploratory and descriptive rather than definitive.

The present findings describe patterns of statistical association within a clinical cohort and should not be interpreted as evidence of mechanistic coupling between structural alignment and neural motor regulation.

## 7. Future Directions

Future research should extend this exploratory multidimensional phenotype-mapping framework to larger, age-stratified cohorts to examine developmental stability and potential trajectory-specific patterns of motor organization in JS. Longitudinal and multicenter designs may help determine whether the structural–functional co-variation observed in the present cohort represents stable regulatory configurations or age-dependent adaptations. Inclusion of typically developing control groups and integration of objective biomechanical, respiratory, and neurophysiological measurements would further enhance interpretability and allow differentiation between syndrome-specific and nonspecific developmental features. In addition, application of model-based or dimensional reduction approaches may clarify whether coherent structural–functional profiles emerge that are clinically meaningful for motor stratification and rehabilitation planning in rare cerebellar disorders. Replication and validation of this multidimensional framework may contribute to the development of structured motor phenotyping approaches applicable across rare cerebellar neurodevelopmental disorders.

## 8. Conclusions

This exploratory study provides a multidimensional characterization of motor phenotype in children with JS within a physiotherapy-based framework. Gross motor performance was associated with postural control, whereas no direct linear relationships were identified between functional outcomes and isolated structural parameters. Structural measures demonstrated internal co-variation but did not independently determine functional capacity. These findings support the relevance of integrated multidomain assessment in clinical evaluation of children with JS and should be interpreted as hypothesis-generating.

## Figures and Tables

**Table 1:** Demographic and Clinical Characteristics of the Cohort.

Variable	Value
Section A. Demographics	
Number of participants, n	25
Age, years, mean ± SD (range)	7.8 ± 4.0 (2–16)
Sex, n (%)	
—Male	16 (64%)
—Female	9 (36%)
Height, cm, mean ± SD (range)	123 ± 20.4 (93–160)
Weight, kg, mean ± SD (range)	26.4 ± 12.2 (11–54)
MRI confirmation (molar tooth sign), n (%)	25 (100%)
Genetic confirmation, n (%)	22 (88%)
Section B. Identified Genotypes	
*TMEM67* (OMIM: 609884)	6
*CEP290* (OMIM: 610142)	3
*DDX41* (OMIM: 608170)	2
*NPHP1* (OMIM: 609583)	2
*AHI1* (OMIM: 608894)	2
*TUBB3* (OMIM: 614039)	1
*PIBF1* (OMIM: 607532)	1
*SUFU* (OMIM: 607035)	1
*NAGA* (OMIM: 104170)	1
*KIAA0586* (OMIM: 616490)	1
*INPP5E* (OMIM: 613037)	1
*MKS1* (OMIM: 617121)	1
Section C. Ocular Features	
Nystagmus	16 (64%)
Strabismus	9 (36%)
Refractive errors	8 (32%)
Oculomotor apraxia	3 (12%)
Retinal dystrophy/retinitis pigmentosa	3 (12%)
Coloboma	1 (4%)
Ptosis	1 (4%)
Severe visual impairment/blindness	1 (4%)
Section D. Renal and Systemic Features	
Any renal involvement	8 (32%)
Kidney transplantation	3 (12%)
Qualification for kidney transplantation	1 (4%)
Renal cysts/cystic kidney disease	3 (12%)
Chronic kidney disease/renal insufficiency	1 (4%)
Liver involvement	3 (12%)
Arterial hypertension	1 (4%)
Gastrointestinal involvement (SIBO)	1 (4%)
Polysomnography performed	8 (32%)
Sleep apnea diagnosed	4 (16%)
Section E. Respiratory Features	
Frequent respiratory infections	12 (48%)
Snoring	8 (32%)
Abnormal breathing sounds	7 (28%)
Sleep apnea/apnea episodes	5 (20%)
Cough	4 (16%)
Hyperventilation	2 (8%)
Other respiratory dysregulation symptoms	4 (16%)

Abbreviations: SD, standard deviation; cm, centimeters; kg, kilograms; %, percentage; MRI, magnetic resonance imaging; n, number of participants; SIBO, small intestinal bacterial overgrowth; OMIM, Online Mendelian Inheritance in Man. Note: Categories were not mutually exclusive.

**Table 2:** Developmental Milestones and Rehabilitation Exposure.

Variable	Value
Section A. Developmental Milestones	
Head control achieved, n (%)	25 (100%)
Age at head control, mean ± SD (range), months	8.7 ± 7.2 (3.5–36)
Independent sitting achieved, n (%)	25 (100%)
Age at independent sitting, mean ± SD (range), months	15.4 ± 8.6 (6.5–36)
Independent standing achieved, n (%)	21 (84%)
Age at independent standing, mean ± SD (range), months	28.7 ± 15.0 (13.5–72)
Independent walking achieved, n (%)	23 (92%)
Age at independent walking, mean ± SD (range), months	36.7 ± 22.3 (17–96)
Never achieved independent standing, n (%)	4 (16%)
Never achieved independent walking, n (%)	2 (8%)
Section B. Rehabilitation Modalities	
NDT Bobath	23 (92%)
Speech therapy	22 (88%)
Sensory integration therapy	21 (84%)
Vision therapy	13 (52%)
Aquatic therapy	13 (52%)
Vojta therapy	10 (40%)
Osteopathy	10 (40%)
Alternative communication therapy	8 (32%)
Functional electrical stimulation	3 (12%)
Respiratory therapy	3 (12%)
Medek therapy	2 (8%)
Reflex integration therapy (INPP/Masgutova)	3 (12%)
Dog-assisted therapy	1 (4%)
Section C. Therapy Frequency	
≥5 therapist-led sessions/week	13 (52%)
3–4 sessions/week	9 (36%)
≤2 sessions/week	3 (12%)
Daily home therapy	6 (24%)
Home therapy ≥ 3×/week	5 (20%)
No regular home therapy	14 (56%)

Abbreviations: SD, standard deviation; %, percentage; NDT, neurodevelopmental treatment; INPP, Institute for Neuro-Physiological Psychology. Note: Developmental milestone ages were based on parent-reported clinical history and converted to months for analysis. Participants who had not achieved a given milestone were excluded from age calculations.

**Table 3:** Descriptive statistics of gross motor function and postural control.

Measure	Mean ± SD	Median	Minimum	Maximum	Shapiro–Wilk W	*p*-Value
GMFM-88 total score (%)	58.4 ± 29.5	78.8	3	89.4	0.796	<0.001
mBARS total score (points)	14.4 ± 9.5	13	2	30	0.897	0.015

Abbreviations: SD, standard deviation; GMFM-88, Gross Motor Function Measure-88; mBARS, modified Brief Ataxia Rating Scale. Note: Shapiro–Wilk test was used to assess normality of distribution. *p*-value < 0.05 indicates significant deviation from normal distribution.

**Table 4:** Musculoskeletal and Postural Parameters.

Parameter	Mean ± SD	Median	Range	Shapiro–Wilk W	*p*-Value
Shoulder ER left (deg)	98.4 ± 27.4	110	20–120	0.691	<0.001
Shoulder ER right (deg)	98.4 ± 27.4	110	20–120	0.691	<0.001
Hip ER left (deg)	65.6 ± 23.3	70	20–90	0.882	0.007
Hip ER right (deg)	63.0 ± 26.6	65	0–90	0.885	0.009
Hip extension right (deg)	16.0 ± 7.6	20	0–20	0.552	<0.001
Hip extension left (deg)	15.6 ± 8.2	20	0–20	0.544	<0.001
Ankle dorsiflexion right (deg)	5.7 ± 8.0	5	−5–28	0.833	<0.001
Ankle dorsiflexion left (deg)	4.2 ± 7.4	0	−5–26	0.745	<0.001
Sacral slope (deg)	16.9 ± 3.8	16	10–25	0.907	0.026

Abbreviations: SD, standard deviation; ER, external rotation; deg, degrees; W, Shapiro–Wilk statistic. Note: Values are presented as mean ± SD, median, and range. Normality of distribution was assessed using the Shapiro–Wilk test. A *p*-value < 0.05 indicates significant deviation from normal distribution.

**Table 5:** Thoracoabdominal Configuration and Respiratory Parameters.

Parameter	Mean ± SD	Median	Range	Shapiro–Wilk W	*p*-Value
Sternoclavicular angle right (deg)	122 ± 11.2	122	105–150	0.933	0.101
Sternoclavicular angle left (deg)	125 ± 9.0	125	105–140	0.950	0.253
Subcostal angle (deg)	119 ± 16.7	120	80–145	0.956	0.348
Chest circumference (cm)	65 ± 11.1	62	52–99	0.888	0.010
Abdominal circumference (cm)	62 ± 11.1	60	49–100	0.834	<0.001
Thoracic expansion (cm)	1.9 ± 1.3	1.5	0–5.5	0.886	0.009
Respiratory rate (breaths/min)	17.2 ± 6.1	18	2–31	0.959	0.388

Abbreviations: SD, standard deviation; cm, centimeters; deg, degrees; breaths/min, breaths per minute; W, Shapiro–Wilk statistic. Note: Values are presented as mean ± SD, median, and range. Normality of distribution was assessed using the Shapiro–Wilk test. A *p*-value < 0.05 indicates significant deviation from normal distribution.

**Table 6:** Statistically Significant Spearman Correlations Between Functional and Structural Parameters.

Variable 1	Variable 2	Spearman ρ	*p*-Value
GMFM-88 total score (%)	mBARS total score (points)	−0.512	0.007
Sacral slope (deg)	Ankle dorsiflexion right (deg)	−0.464	0.019
Sacral slope (deg)	Ankle dorsiflexion left (deg)	−0.456	0.022
Sacral slope (deg)	Sternoclavicular angle left (deg)	0.457	0.021
Sacral slope (deg)	Chest circumference (cm)	0.724	<0.001
Sacral slope (deg)	Abdominal circumference (cm)	0.530	0.006
Sacral slope (deg)	Hip extension right (deg)	−0.614	0.001
Sacral slope (deg)	Hip extension left (deg)	−0.629	<0.001

Abbreviations: GMFM-88, Gross Motor Function Measure-88; mBARS, modified Brief Ataxia Rating Scale; deg, degrees; cm, centimeters; ρ, Spearman’s rank correlation coefficient. Note: Spearman’s rank correlation coefficients were calculated between predefined functional and structural.

**Table 7:** Intra-Rater Reliability and Measurement Error of Selected Parameters.

Parameter	ICC (3,1)	95% CI	*p*-Value	SEM	MDC95
Sacral slope	0.939	0.866–0.973	<0.001	0.93°	2.58°
Subcostal angle	0.996	0.991–0.998	<0.001	—	—
Thoracic expansion	0.979	0.953–0.991	<0.001	0.20 cm	0.55 cm
Ankle dorsiflexion (right)	0.996	0.990–0.998	<0.001	0.51°	1.41°
Ankle dorsiflexion (left)	0.996	0.991–0.998	<0.001	—	—
Hip ER (right)	0.999	0.998–1.000	<0.001	—	—
Hip ER (left)	0.999	0.998–1.000	<0.001	—	—
Hip extension (right)	0.986	0.964–0.994	<0.001	—	—
Hip extension (left)	0.991	0.976–0.996	<0.001	—	—
Shoulder ER (right)	0.998	0.994–0.999	<0.001	—	—
Shoulder ER (left)	0.998	0.996–0.999	<0.001	—	—
Chest circumference	0.999	0.997–0.999	<0.001	—	—
Abdominal circumference	0.989	0.976–0.995	<0.001	—	—
Sternoclavicular angle (right)	0.981	0.957–0.991	<0.001	—	—
Sternoclavicular angle (left)	0.964	0.919–0.984	<0.001	—	—
Respiratory rate	0.989	0.976–0.995	<0.001	—	—

Abbreviations: ICC, intraclass correlation coefficient; CI, confidence interval; SEM, standard error of measurement; MDC95, minimal detectable change at the 95% confidence level; ER, external rotation. Note: ICC (3,1) was calculated using a two-way mixed-effects model with absolute agreement. Measurements were repeated within the same session by the same examiner. SEM and MDC95 were calculated for selected representative parameters.

## Data Availability

The data presented in this study are not publicly available due to ethical and privacy restrictions. Anonymized data may be made available from the corresponding author upon reasonable request.

## Supplementary Materials

The following supporting information can be downloaded at: https://www.mdpi.com/article/10.3390/jcm15093221/s1, Figure S1: Bland–Altman plot for right ankle dorsiflexion; Figure S2: Bland–Altman plot for sacral slope; Figure S3: Bland–Altman plot for thoracic expansion; Table S1: Full Spearman correlation matrix for predefined functional and structural variables.

## Abbreviations

The following abbreviations are used in this manuscript:

JS	Joubert Syndrome
GMFM-88	Gross Motor Function Measure-88
mBARS	modified Brief Ataxia Rating Scale
MRI	Magnetic Resonance Imaging
SD	Standard Deviation
CI	Confidence Interval
ICC	Intraclass Correlation Coefficient
SEM	Standard Error of Measurement
MDC95	Minimal Detectable Change at the 95% confidence level
ER	External Rotation
deg	Degrees
cm	Centimeters
kg	Kilograms
N	Number of participants
%	Percentage
W	Shapiro–Wilk statistic
*p*	Spearman’s rank correlation coefficient
breaths/min	Breaths per minute
SIBO	Small Intestinal Bacterial Overgrowth
NDT	Neurodevelopmental Treatment
INPP	Institute for Neuro-Physiological Psychology
OMIM	Online Mendelian Inheritance in Man

## References

[1] Bachmann-Gagescu R., Dempsey J.C., Bulgheroni S., Chen M.L., D’Arrigo S., Glass I.A., Heller T., Héon E., Hildebrandt F., Joshi N. (2020). Healthcare Recommendations for Joubert Syndrome. Am. J. Med. Genet. Part A.

[2] Gurbani S.S., Assaye T., D’souza T.X., Dennison J.V., Palasis S., Bajaj M. (2025). Beyond the Molar Tooth: A Review of Imaging Findings in Joubert Syndrome and Related Disorders. Neurographics.

[3] Owens J.W., Hopkin R.J., Martin L.J., Kodani A., Simpson B.N. (2024). Phenotypic Variability in Joubert Syndrome Is Partially Explained by Ciliary Pathophysiology. Ann. Hum. Genet..

[4] Serpieri V., D’Abrusco F., Valente E.M. (2025). The Relevance of Primary Cilia in Neurological Disorders. Lancet Neurol..

[5] Tran A.M., Jnah A.J., De Castro Pretelt M.J. (2025). Genetics Review: Joubert Syndrome. Neonatal Netw..

[6] Romani M., Micalizzi A., Valente E.M. (2013). Joubert Syndrome: Congenital Cerebellar Ataxia with the “Molar Tooth”. Lancet Neurol..

[7] Poretti A., Huisman T.A.G.M., Scheer I., Boltshauser E. (2011). Joubert Syndrome and Related Disorders: Spectrum of Neuroimaging Findings in 75 Patients. AJNR Am. J. Neuroradiol..

[8] Brancati F., Dallapiccola B., Valente E.M. (2010). Joubert Syndrome and Related Disorders. Orphanet J. Rare Dis..

[9] Weghe J.C.V.D., Gomez A., Doherty D. (2022). The Joubert–Meckel–Nephronophthisis Spectrum of Ciliopathies. Annu. Rev. Genom. Hum. Genet..

[10] Alzarka B., Charnaya O., Gunay-Aygun M. (2025). Diseases of the Primary Cilia: A Clinical Characteristics Review. Pediatr. Nephrol..

[11] Szewczyk A.K., Papuć E., Mitosek-Szewczyk K., Woś M., Rejdak K. (2022). NMOSD—Diagnostic Dilemmas Leading towards Final Diagnosis. Brain Sci..

[12] Walkowiak D., Domaradzki J. (2021). Are Rare Diseases Overlooked by Medical Education? Awareness of Rare Diseases among Physicians in Poland: An Explanatory Study. Orphanet J. Rare Dis..

[13] Parisi M.A. (2019). The Molecular Genetics of Joubert Syndrome and Related Ciliopathies: The Challenges of Genetic and Phenotypic Heterogeneity. Transl. Sci. Rare Dis..

[14] Bulgheroni S., D’Arrigo S., Signorini S., Briguglio M., Di Sabato M.L., Casarano M., Mancini F., Romani M., Alfieri P., Battini R. (2016). Cognitive, Adaptive, and Behavioral Features in Joubert Syndrome. Am. J. Med. Genet. Part A.

[15] Gana S., Serpieri V., Valente E.M. (2022). Genotype-Phenotype Correlates in Joubert Syndrome: A Review. Am. J. Med. Genet. Part C Semin. Med. Genet..

[16] Proske U., Gandevia S.C. (2012). The Proprioceptive Senses: Their Roles in Signaling Body Shape, Body Position and Movement, and Muscle Force. Physiol. Rev..

[17] da Silva K.M., Pádua R.F., Sá C.d.S.C.d., Carvalho R.d.P. (2024). Relationship between Trunk Control and Gross Motor Development of Infants in the First Year of Life: A Systematic Review. Early Hum. Dev..

[18] Rico-González M., Gómez-Carmona C.D., Ouergui I., Ardigò L.P. (2025). Machine Learning Methods in Posture-Related Applications in Children up to 12 Years Old: A Systematic Review. Bioengineering.

[19] Assaiante C., Mallau S., Viel S., Jover M., Schmitz C. (2005). Development of Postural Control in Healthy Children: A Functional Approach. Neural Plast..

[20] Sanger T.D., Chen D., Delgado M.R., Gaebler-Spira D., Hallett M., Mink J.W. (2006). Taskforce on Childhood Motor Disorders Definition and Classification of Negative Motor Signs in Childhood. Pediatrics.

[21] Katalkar P., Iyer L., Walankar P.P., Karnik S. (2026). Trunk Repositioning Sense, Balance, and Reaction Time, in People with Attention Deficit Hyperactivity Disorder: A Cross-Sectional Study. Bull. Fac. Phys. Ther..

[22] Glass I.A., Dempsey J.C., Parisi M., Doherty D., Adam M.P., Bick S., Mirzaa G.M., Pagon R.A., Wallace S.E., Amemiya A. (1993). Joubert Syndrome. GeneReviews^®^.

[23] Summers A.C., Snow J., Wiggs E., Liu A.G., Toro C., Poretti A., Zein W.M., Brooks B.P., Parisi M.A., Inati S. (2017). Neuropsychological Phenotypes of 76 Individuals with Joubert Syndrome Evaluated at a Single Center. Am. J. Med. Genet. Part A.

[24] Stucki G., Melvin J. (2007). The International Classification of Functioning, Disability and Health: A Unifying Model for the Conceptual Description of Physical and Rehabilitation Medicine. J. Rehabil. Med..

[25] Mański Ł., Moluszys A., Góra A., Wasilewska E., Rosa A., Szczałuba K., Szymańska K., Wierzba J. (2026). Multidimensional Functional Phenotyping in Children with Joubert Syndrome: A Pilot Case Series. Brain Sci..

[26] Mański Ł., Moluszys A., Wasilewska E., Rosa A., Szczałuba K., Szumlicki J., Szymańska K., Wierzba J. (2026). Neurorehabilitation and Functional Improvement in Joubert Syndrome: A 12-Month Case Report. Children.

[27] Mański Ł., Moluszys A., Wierzba J. (2026). Functional Motor Assessment and Rehabilitation in Joubert Syndrome: A Narrative Review and Conceptual Framework for Pediatric Neurorehabilitation. Children.

[28] Maria B.L., Boltshauser E., Palmer S.C., Tran T.X. (1999). Clinical Features and Revised Diagnostic Criteria in Joubert Syndrome. J. Child Neurol..

[29] Apps R., Garwicz M. (2005). Anatomical and Physiological Foundations of Cerebellar Information Processing. Nat. Rev. Neurosci..

[30] Shahshahani L., King M., Nettekoven C., Ivry R.B., Diedrichsen J. (2024). Selective Recruitment of the Cerebellum Evidenced by Task-Dependent Gating of Inputs. eLife.

[31] Ito M. (2008). Control of Mental Activities by Internal Models in the Cerebellum. Nat. Rev. Neurosci..

[32] Manto M. (2008). The Cerebellum, Cerebellar Disorders, and Cerebellar Research—Two Centuries of Discoveries. Cerebellum.

[33] Morton S.M., Bastian A.J. (2004). Cerebellar Control of Balance and Locomotion. Neurosci. Rev. J. Bringing Neurobiol. Neurol. Psychiatry.

[34] Curtis D.J., Butler P., Saavedra S., Bencke J., Kallemose T., Sonne-Holm S., Woollacott M. (2015). The Central Role of Trunk Control in the Gross Motor Function of Children with Cerebral Palsy: A Retrospective Cross-Sectional Study. Dev. Med. Child Neurol..

[35] Saavedra S.L., Woollacott M.H. (2015). Segmental Contributions to Trunk Control in Children with Moderate-to-Severe Cerebral Palsy. Arch. Phys. Med. Rehabil..

[36] Giavarina D. (2015). Understanding Bland Altman Analysis. Biochem. Medica.

[37] Achonu J.U., Ling K., Bhan R., Garcia A., Komatsu D.E., Pallotta N.A. (2023). Pelvic Index: A New Pelvic Parameter for Assessing Sagittal Spinal Alignment. N. Am. Spine Soc. J..

[38] Chen X., Shi H.-G., Wan D., Deng X.-G., Song S.-M., Cao M. (2023). Relationship between Lumbar Disc Herniation and Roussouly Classification in the Sagittal Alignment of the Spine and Pelvis in Young People. Quant. Imaging Med. Surg..

[39] Horak F.B., Nashner L.M. (1986). Central Programming of Postural Movements: Adaptation to Altered Support-Surface Configurations. J. Neurophysiol..

[40] Krohn F., Novello M., van der Giessen R.S., De Zeeuw C.I., Pel J.J.M., Bosman L.W.J. (2023). The Integrated Brain Network That Controls Respiration. eLife.

[41] Moreira T.S., Takakura A.C., Falquetto B., Ramirez J.-M., Oliveira L.M., Silva P.E., Araujo E.V. (2025). Neuroanatomical and Neurochemical Organization of Brainstem and Forebrain Circuits Involved in Breathing Regulation. J. Neurophysiol..

[42] Hodges P.W., Heijnen I., Gandevia S.C. (2001). Postural Activity of the Diaphragm Is Reduced in Humans When Respiratory Demand Increases. J. Physiol..

[43] Hodges P.W., Gandevia S.C. (2000). Changes in Intra-Abdominal Pressure during Postural and Respiratory Activation of the Human Diaphragm. J. Appl. Physiol..

[44] Hodges P.W., Eriksson A.E.M., Shirley D., Gandevia S.C. (2005). Intra-Abdominal Pressure Increases Stiffness of the Lumbar Spine. J. Biomech..

[45] Florio T.M. (2025). Emergent Aspects of the Integration of Sensory and Motor Functions. Brain Sci..

[46] Kakei S., Lee J., Mitoma H., Tanaka H., Manto M., Hampe C.S. (2019). Contribution of the Cerebellum to Predictive Motor Control and Its Evaluation in Ataxic Patients. Front. Hum. Neurosci..

[47] Konosu A., Matsuki Y., Fukuhara K., Funato T., Yanagihara D. (2024). Roles of the Cerebellar Vermis in Predictive Postural Controls against External Disturbances. Sci. Rep..

[48] Farinelli V., Palmisano C., Marchese S., Strano C., D’Arrigo S., Pantaleoni C., Ardissone A., Nardocci N., Esposti R., Cavallari P. (2020). Postural Control in Children with Cerebellar Ataxia. Appl. Sci..

[49] Novak I., McIntyre S., Morgan C., Campbell L., Dark L., Morton N., Stumbles E., Wilson S.-A., Goldsmith S. (2013). A Systematic Review of Interventions for Children with Cerebral Palsy: State of the Evidence. Dev. Med. Child Neurol..

