# Supplementary material for: Multidimensional Motor Phenotype Characterization in Children with Joubert Syndrome: A Cross-Sectional Cohort Study
Source: J Clin Med. 2026 Apr 23;15(9):3221. doi: 10.3390/jcm15093221 (PMC13163950; doi:10.3390/jcm15093221)
Supplement: Supplementary file 1 [file jcm-15-03221-s001.zip › Supplementary Figures S1-S3.pdf]

Supplementary Figures

Bland–Altman plots illustrating intra-rater agreement for selected representative parameters assessed within the multidimensional assessment framework.

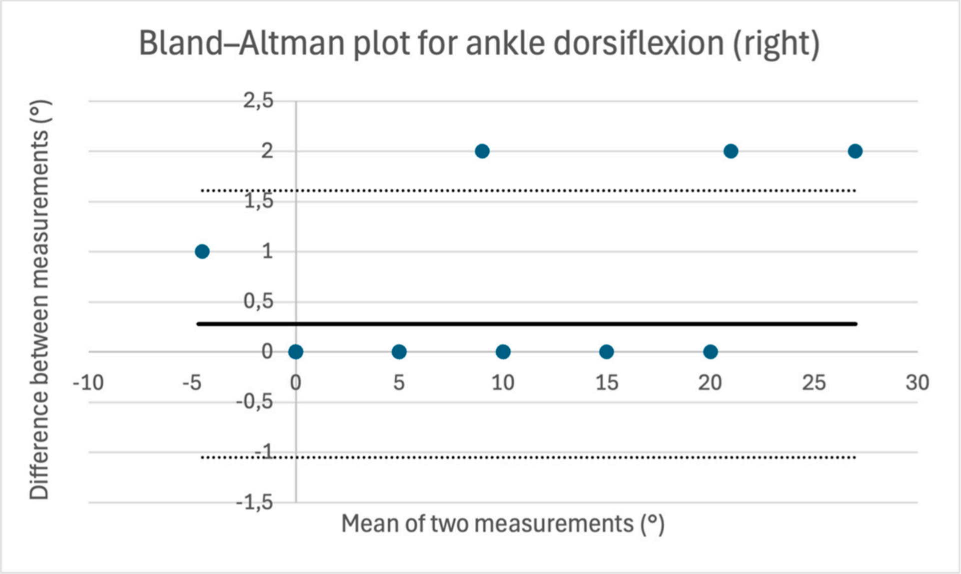

Figure S1. Bland–Altman plot for right ankle dorsiflexion (degrees).
The solid horizontal line represents the mean difference (bias) between repeated measurements. The dotted lines indicate the 95% limits of agreement (mean difference ± 1.96 SD). Each point represents one participant (n = 25). The distribution of differences suggests acceptable within-session agreement without evidence of systematic proportional bias.

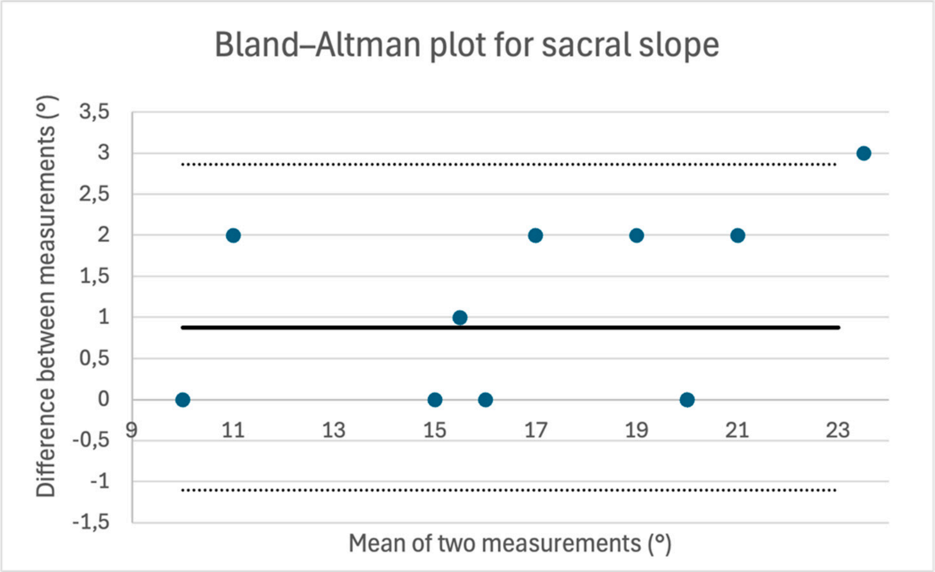

Figure S2. Bland–Altman plot for sacral slope (degrees).
The solid horizontal line represents the mean difference (bias) between repeated measurements. The dotted lines indicate the 95% limits of agreement (mean difference ± 1.96 SD). Each point represents one participant (n = 25). No apparent proportional bias was observed across the measurement range.

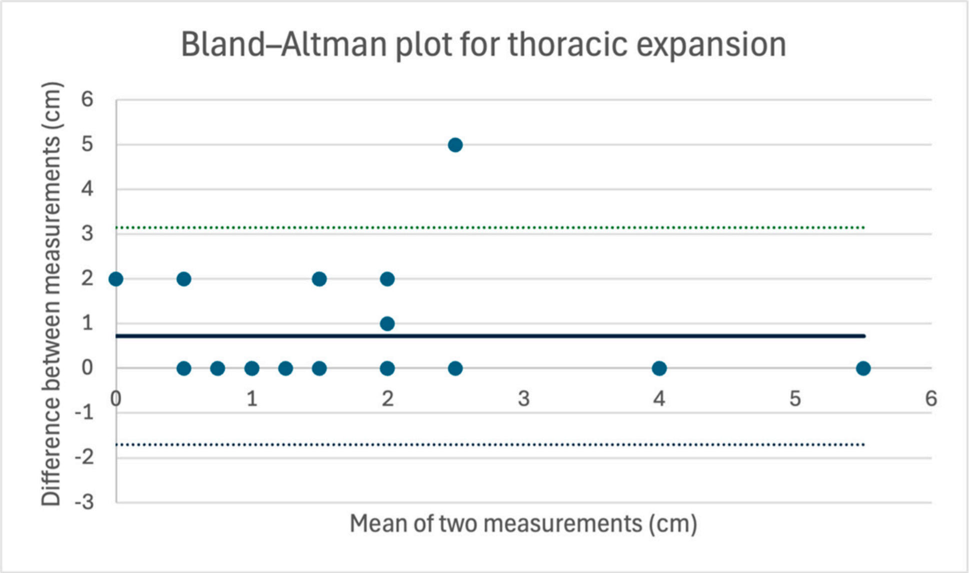

Figure S3. Bland–Altman plot for thoracic expansion (cm).

The solid horizontal line represents the mean difference (bias) between repeated measurements. The dotted lines indicate the 95% limits of agreement (mean difference ± 1.96 SD). Each point represents one participant (n = 25). Limits of agreement were narrow relative to the observed measurement range.
