# Supplementary material for: Multidimensional Motor Phenotype Characterization in Children with Joubert Syndrome: A Cross-Sectional Cohort Study
Source: J Clin Med. 2026 Apr 23;15(9):3221. doi: 10.3390/jcm15093221 (PMC13163950; doi:10.3390/jcm15093221)
Supplement: Supplementary file 1 [file jcm-15-03221-s001.zip › Supplementary Table S1.pdf]

Supplementary Materials

Supplementary Table S1. Full Spearman Correlation Matrix for Predefined Functional and Structural Variables in Children with Joubert Syndrome (n = 25)

Variable	1	2	3	4	5	6	7	8	9	10
1. GMFM_%	—									
2. BARS_total	−0.514 (0.009)	—								
3. Sacral slope	0.215 (0.303)	−0.170 (0.416)	—							
4. Hip ext R	0.217 (0.299)	−0.324 (0.114)	−0.363 (0.074)	—						
5. Hip ext L	0.160 (0.446)	−0.278 (0.178)	−0.553 (0.004)	0.968 (<0.001)	—					
6. Ankle DF R	0.301 (0.144)	0.070 (0.739)	−0.444 (0.026)	0.211 (0.311)	0.237 (0.254)	—				
7. Ankle DF L	0.067 (0.751)	0.212 (0.308)	−0.444 (0.026)	0.055 (0.795)	0.166 (0.428)	0.899 (<0.001)	—			
8. Sternoclavicular L	0.152 (0.469)	0.036 (0.865)	0.450 (0.024)	0.261 (0.207)	0.277 (0.180)	−0.185 (0.376)	−0.140 (0.505)	—		
9. Chest circumference	0.405 (0.045)	−0.046 (0.828)	0.765 (<0.001)	−0.537 (0.006)	−0.498 (0.011)	−0.391 (0.053)	−0.397 (0.050)	0.356 (0.081)	—	
10. Abdominal circumference	0.282 (0.171)	−0.103 (0.623)	0.597 (0.002)	−0.415 (0.039)	−0.409 (0.043)	−0.080 (0.705)	−0.073 (0.729)	0.481 (0.015)	0.830 (<0.001)	—

Abbreviations: GMFM, Gross Motor Function Measure-88; BARS, modified Brief Ataxia Rating Scale; Hip ext, hip extension; DF, dorsiflexion; R, right; L, left
Note: Spearman's rank correlation coefficients (ϱ) are presented with corresponding p-values in parentheses. Analyses were conducted between predefined functional and structural variables according to the multidimensional assessment framework. Correlation analyses were conducted within an exploratory framework and were not adjusted for multiple comparisons; findings should therefore be interpreted as hypothesis-generating rather than confirmatory.
